# Nitrogen and hydrogen as carrier and make‐up gases for GC‐MS with Cold EI

**DOI:** 10.1002/jms.4830

**Published:** 2022-04-26

**Authors:** Ksenia J. Margolin Eren, Harry Frank Prest, Aviv Amirav

**Affiliations:** ^1^ School of Chemistry Tel Aviv University Tel Aviv Israel; ^2^ Mountain View California USA; ^3^ Aviv Analytical Ltd Hod Hasharon Israel

**Keywords:** Cold EI, GC column carrier gas, GC‐MS, hydrogen carrier gas, interrupted helium supply, nitrogen carrier gas, supersonic molecular beams

## Abstract

Gas chromatography–mass spectrometry (GC‐MS) with Cold EI is based on interfacing GC and MS with a supersonic molecular beam (SMB) and sample compounds ionization with a fly‐through ion source as vibrationally cold compounds in the SMB (hence the name Cold EI). We explored the use of nitrogen and hydrogen as carrier and make‐up gases with Cold EI and found:

- Nitrogen is very effective in cooling compounds in SMB and while helium requires 60 ml/min nitrogen provides effective cooling with only 7–8 ml/min combined column and make‐up flow rate. Hydrogen is less effective than helium and requires higher flow rates.
- The transition from helium to nitrogen (or hydrogen) is simple and fast and requires just closing the helium valve and opening the nitrogen valve.
- The same column used with helium can be used with nitrogen or hydrogen.
- The same elution times could be obtained with nitrogen or hydrogen as with helium.
- The GC separation with nitrogen was reduced compared with helium and peak widths were increased by an average factor of 1.5 for similar elution times. Hydrogen provided ~0.7 narrower peak widths than helium.
- The signal with nitrogen was reduced compared with helium by an average factor of 3.3 and the signal loss was reduced with higher compounds mass. With hydrogen the signal loss was about a factor of 1.5 but the baseline noise was higher thus with similar S/N as with nitrogen.
- USEPA 8270 semivolatile mixture was easily analyzed with both nitrogen and hydrogen carrier gases.

## 1 INTRODUCTION

The subject of helium shortages or its interrupted supply has led to investigations of hydrogen or nitrogen as alternative carrier gases for gas chromatography–mass spectrometry (GC‐MS), which have been discussed in papers,^1^, ^2^, ^3^, ^4^, ^5^, ^6^, ^7^ blog discussions,^8^ and GC‐MS vendor seminars and presentations.^9^, ^10^, ^11^, ^12^, ^13^, ^14^ Different attempts for helium conservation method and its related instrument development were also made.^9^, ^15^ However:

1. **There is no intrinsic helium shortage.** We never experienced any helium supply problem and believe that the helium shortage problem if exists is not a long lasting one as alternative natural gas wells are being found with very high helium amounts^16^, ^17^ and new deep drill gas wells are likely to contain helium due to long time radioactivity in these wells. Furthermore, helium is found at 5.2 ppm in air. Thus, due to the advantages of using helium our advice is to continue its use as the carrier gas for GC‐MS while purchasing additional spare cylinders “just in case” that a temporal shortage will occur and induce short term supply interruption.
2. **Helium gas is not expensive.** Currently the price of raw helium is $3.1 per cubic meter (in 2020) to US government users and $4.29 for others^18^ and the price of 99.999% helium at GC‐MS laboratories is around $30 per cubic meter ($20 in the USA). Thus, even if the price of raw helium will increase up to five times, its price to end users may only be doubled because most of the price is due to the helium purification and shipment. Typical GC‐MS consumes about 15–20 ml/min mostly via the injector split and septum purge, as most of the time it is in the gas saver mode or with split ratio below 20. Thus, for 40 min analysis time, the helium consumption is under 0.8 L that costs 2.4 cents, which is negligible compared with other costs of the analysis such as operator salary, system amortization, electricity, city tax, maintenance, and other consumables. While GC‐MS with Cold EI consumes 50 ml/min helium as make‐up gas for generating a “cold beam” typical analysis times are below 10 min and, with GC oven cooling, it is in total about 14 min. Thus, at 70 ml/min total GC‐MS with Cold EI helium consumption per minute, the helium consumption per analysis is 1 L with cost of 3 cents, which is negligible. At night or “stand‐by modes,” the helium consumption is under 10 ml/min in both standard EI and Cold EI. Thus, one helium cylinder with 10 m^3^ that costs $300 serves one GC‐MS system for a year (if there are no leaks) at the price of under $1/day. We note that the solvents price of liquid chromatography–mass spectrometry (LC‐MS) in their ultra‐high performance liquid chromatography–mass spectrometry (UHPLC‐MS) grade cost over 10 times the price of helium in GC‐MS per analysis, yet little has been written that it is too expensive.

However, if and when the helium supply is disrupted, hydrogen or nitrogen can serve as easily obtained alternative carrier gases, but they do come with their own set of problems. Hydrogen is a highly reactive gas and can degrade certain compounds without a simple rule as to which compounds will be most susceptible.^13^, ^19^, ^20^ This hydrogen‐related reactivity occurs mostly via three reasons: (a) Direct homogeneous reactions such as the reduction of nitro groups in explosives and other nitro compounds; (b) indirect catalytic decomposition via the promotion of surface activity at the GC injector liner, which becomes reactive with exposed reactive silanol groups and reduced metal oxides into bare metals that promote catalytic decomposition; and (c) similarly, the standard EI ion source metallic surfaces become clean and thus “activated” via the hydrogen reactions with its surface constituents and consequently the exposed metal is reactive to several classes of compounds. Thus, ion source‐related activity reduces the active compounds response and also changes the mass spectral fragmentation pattern, which impedes the ability to perform library‐based identification. Accurate quantitation is also compromised because “conversions” can alter target compound quantitation and confirming ions. Cold EI suffers from the first two problems of hydrogen but not from any ion source‐related reactivity in view of its contact‐free fly‐through ion source structure. Hydrogen also somewhat reduces the sensitivity of standard EI ion sources, and gas viscosity considerations require lower head pressures for comparable or optimal flows with hydrogen. So to lower the flow to the ion source and meet the chromatographic demands, hydrogen requires the use of a smaller I.D. column with its related problems of lower column capacity, linear dynamic range (LDR), and lifetime. Furthermore, hydrogen generators are expensive (cost $5000–10,000) and require their own maintenance, and hydrogen usage can generate safety problems, which often requires costly compliance with local safety regulations. Thus, while promoted by certain hydrogen generators producers and GC‐MS vendors, the use of hydrogen is undesirable as it reduces the range of compounds and applications amenable for GC‐MS analysis and greatly increases the complexity of GC‐MS applications.

Accordingly, the best alternative to helium as a GC‐MS carrier gas is nitrogen (N_2_). However, while nitrogen is relatively inert, its most salient downside is that it significantly reduces the GC‐MS sensitivity in standard EI. The main reason for this sensitivity reduction is that N_2_ ionization yield is approximately nine times higher than of helium and thus the intra‐ion‐source space‐charge is very high, equivalent to the use of 9 ml/min helium flow rate versus 1 ml/min. Standard EI ion sources are not adapted for such high intra‐ion‐source space‐charge, which results in a major sensitivity reduction by a factor of more than 20. Furthermore, nitrogen as a carrier gas requires for optimal separation a slower temperature program rate; hence, it requires either longer analysis times than with helium or some compromise with reduced separation. These nitrogen carrier gas problems are partially addressed via the use of smaller column diameters such as 0.15‐mm I.D. that are operated with only 0.3 ml/min column flow rate but at the price of lower column capacity, LDR, and lifetime. Therefore, the first solution for the possible problem of helium shortage is to have additional spare helium cylinders while the use of nitrogen should also be explored for “just in case.”

This paper presents and describes a viable approach to avoid any helium shortage problem with GC‐MS with Cold EI through using nitrogen as both the GC column carrier gas and cooling make‐up gas. We also explore the use of hydrogen for comparison. Cold EI was initially developed in 1990,^21^, ^22^ it is reviewed in Amirav et al.,^23^, ^24^ and a book on GC‐MS with Cold EI was recently published.^25^ GC‐MS with Cold EI is based on interfacing the GC and MS with supersonic molecular beams (SMB) along with electron ionization of vibrationally cold sample compounds in the SMB in a fly‐through ion source (hence the name Cold EI). GC‐MS with Cold EI improves all the central performance aspects of GC‐MS: Enhanced molecular ions, improved sample identification, significantly extended range of compounds amenable for analysis, uniform response to all analytes, faster analysis, greater selectivity, and lower limits of detection. We already explored in the past and described the use of nitrogen as the GC carrier gas in GC‐MS with Cold EI while hydrogen was used as the cooling make‐up gas^26^ while providing similar sensitivity as with helium. However, for simplicity, it is better to be prepared with only one type of helium alternative gas, which we suggest should be nitrogen as described in this manuscript.

## 2 EXPERIMENTAL

We used the 5975‐SMB GC‐MS with Cold EI system that is based on the combination of an Agilent 7890A GC + 5975B MSD from 2009 (Agilent Technologies, Santa Clara CA USA) with the Aviv Analytical supersonic molecular beam interface and its dual‐cage fly‐through ion source (Aviv Analytical LTD, Hod Hasharon Israel). The technology of GC‐MS with Cold EI is reviewed in Amirav et al.^23^, ^24^ and fully described in a recent book.^25^

In GC‐MS with Cold EI, the GC column output is mixed with helium or nitrogen or hydrogen make‐up gas (~60 ml/min typical total column and make‐up helium or hydrogen flow rate combined or typically 8 ml/min total column and make‐up nitrogen flow rate combined), in front of a supersonic nozzle located at the end of a heated, temperature ramped transfer line. The helium or nitrogen or hydrogen make‐up gas flow can be mixed (via the opening of one valve) with perfluorotributylamine (PFTBA) for periodic system tuning and mass calibration. The sample compounds seeded in the helium or nitrogen or hydrogen gas expand from a 100‐μm diameter supersonic nozzle into an SMB nozzle vacuum chamber that is differentially pumped by a Varian Navigator 301 turbo molecular pump (Varian Inc., Torino Italy) with 250 L/s pumping speed. The supersonic expansion vibrationally cools the sample compounds, and the expanded supersonic free jet is sampled (skimmed) by a 0.8‐mm diameter hole on a cone‐shaped skimmer and collimated in a second differentially pumped vacuum chamber, where the analytical SMB is formed. The second vacuum chamber is pumped by the Agilent 5975 system “Performance” turbo molecular pump that pumps the dual‐cage fly‐through ion source and MS (Pfeiffer 250 L/s pump). The SMB containing vibrationally cold sample compounds passes the fly‐through dual‐cage EI ion source^27^ where these beam species are ionized by 70‐eV electrons at 6‐mA emission current. It is worthwhile noting that the Cold EI ion source has been used for over 10 years while maintaining almost the same performance without any service except for one filament replacement. The ions are focused by an ion lens system, deflected 90° by an ion mirror, and enter the Agilent 5975B MS for their mass analysis. The 90° ion mirror is separately heated and serves to keep the mass analyzer clean from possible sample induced contaminations. The ions that exit the Agilent MS are detected by the Agilent Triple Axis ion detector and the data is processed by the Agilent Chemstation software.

GC separation was performed with a 15‐m column with 0.32‐mm I.D., 0.1‐μm DB1‐HT film (Agilent Technologies Folsom CA USA) with typically 2 up to 8 ml/min column flow rate. The GC oven temperature was ramped from 50°C to 300°C at 40°C/min and held at 300°C for 1.75 min. A standard test mixture of 10 ng/μl each n‐C_16_H_32_, methyl stearate, cholesterol, and n‐C_32_H_66_ was analyzed. This sample was injected split at a 4:1 ratio to provide 2 ng each compound on column. OFN was used as provided by Agilent at 1 pg/μl with split 9:1 thus 100 fg on‐column. A USEPA 8270 test mixture was used as purchased from Restek (Restek Bellefonte PA USA part number 31687) at 100 ng/μl each compound and injected with split ratio 20 to have 5 ng for each compound on‐column. Nitrogen gas was provided from a 10‐m^3^ cylinder at 99.999 purity via copper tubing and only its make‐up gas was filtered by a carbon gas filter. Hydrogen was provided by a Domnick Hunter (Gateshead, UK) hydrogen generator, and similarly, only its make‐up gas was filtered by a carbon gas filter.

Analytical conditions summary:

**System:** Aviv Analytical 5975‐SMB GC‐MS with Cold EI.

**Gases:** Helium in a cylinder at 99.999% purity or Nitrogen in a cylinder at 99.999% purity or hydrogen as provided by its generator.

**Injector:** 250°C injector temperature.

**Liner:** Jennings cup (Agilent technologies part number 18740–80190).

**Sample:** Aviv Analytical standard test mixture of 10 ng/μl each hexadecane (n‐C_16_H_34_), methyl stearate, cholesterol, and n‐C_32_H_66_ in hexane. The sample was originally purchased from Restek at 1000 ng/μl and diluted to 10 ng/μl. USEPA 8270 semivolatile test mixture was used as purchased from Restek, and OFN was used as provided by Agilent in a 1 pg/μl vial.

**Column:** 15 m 0.32 mm ID, 0.1 μm film of DB1‐HT.

**Column flow rate:** 8 ml/min He in the helium carrier gas experiments and 2–15 ml/min N_2_ in the nitrogen carrier gas experiments and 2–5 ml/min in the hydrogen carrier gas experiments.

**Make up gas flow rate:** 52–58 ml/min He or H_2_ flow rate in the helium and hydrogen carrier gases experiments and 0–12 ml/min in the nitrogen carrier gas experiments.

**Injection volume:** 1 μl.

**Split ratio:** Split 5 or 10 or 20.

**On column amounts:** 2 ng each of the test mixture sample compounds, 5‐ng 8270 mixture compounds and 100‐fg OFN.

**GC Oven:** 50°C followed by 40°C/min to 300°C and hold 1.75 min for total of 8 min for the test mixture analysis. 40°C for 2 min followed by 20°C/min to 300°C for a total of 15 min with hydrogen gas for the analysis of the 8270 mixture. Nitrogen was the same as for hydrogen but without the 2 min hold for a total of 13 min. For OFN analysis with hydrogen and nitrogen, it was 40°C for 0.25 min then 40°C/min to 190°C for a total of 4 min.

**Cold EI source:** 6 mA emission current with all three gases.

**Electron Energy:** 70 eV.

**SMB transferline temperature:** 250°C with temperature program of 10°C/min after 4 min to 270°C.

**MS parameters:** Mass spectral scan range was 50–500 amu and scan speed ~3.2 Hz. In OFN, it was single ion monitoring on m/z = 272 with 300 ms dwell time.

## 3 RESULTS

### 3.1 GC‐MS with Cold EI Operation with Nitrogen

**FIGURE 1:**
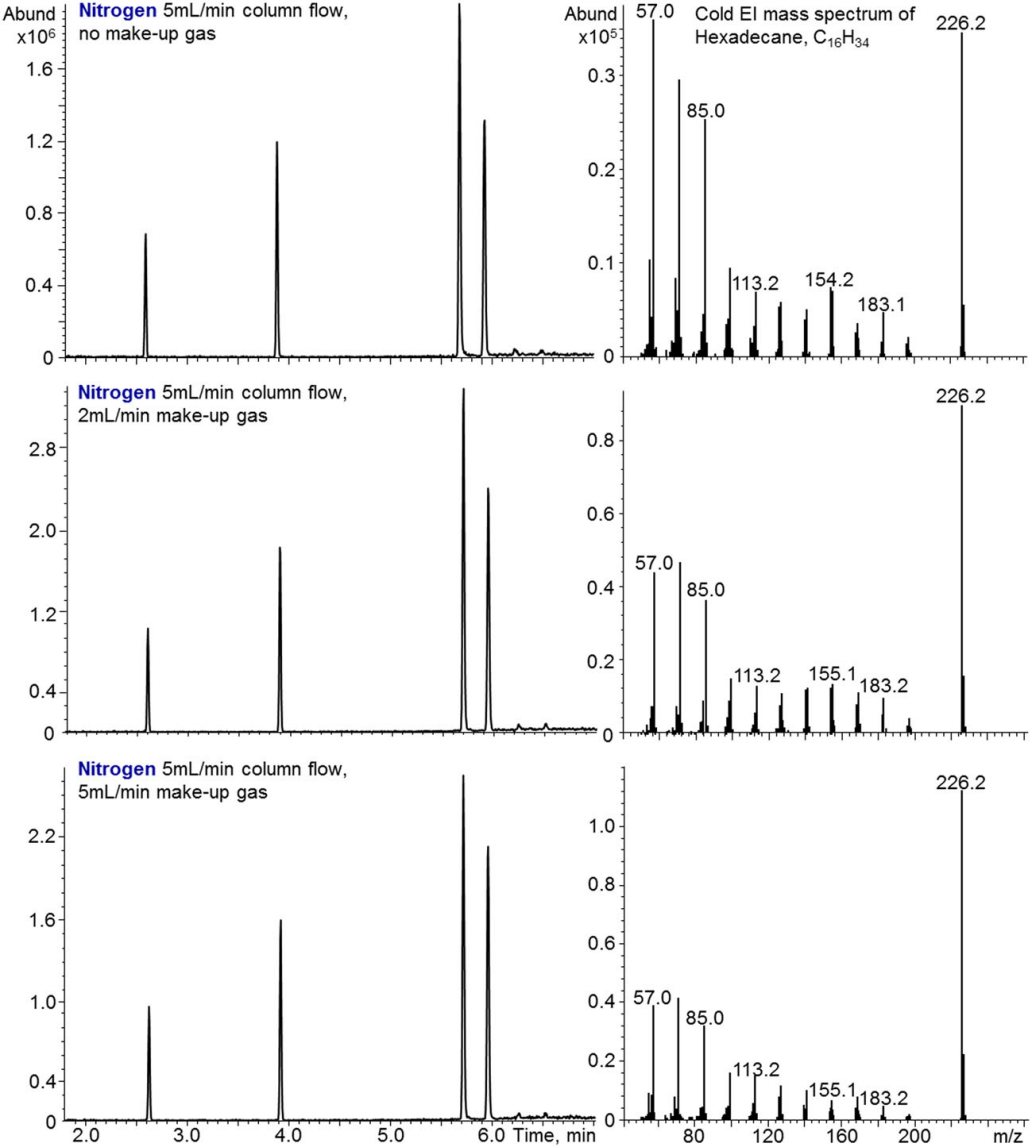
The effect of nitrogen make‐up gas flow rate on the analysis of a test mixture of 2 ng each n‐C_16_H_32_, methyl stearate, cholesterol, and n‐C_32_H_66_. The nitrogen carrier gas flow rate was 5 ml/min, whereas the nitrogen make‐up flow rates were as indicated 0, 2, and 5 ml/min for total nitrogen cooling flow rates from the supersonic nozzle of 5, 7, and 10 ml/min. The resulting Cold EI mass spectra of n‐C_16_H_34_ are shown at right. Note the changes in the ratio of molecular ion to m/z = 57 fragment ion

The initial GC‐MS with Cold EI experiments with nitrogen were to characterize the vibrational cooling efficiency per given total column and make‐up flow rate. In Figure 1, we show that we can easily analyze our standard test mixture that includes 2 ng each hexadecane (n‐C_16_H_34_), methyl stearate, cholesterol, and dotriacontane (n‐C_32_H_66_) in order of their elution times. As demonstrated, even at 5 ml/min combined column and make‐up gas flow rates, we obtain noticeable cooling, whereas at 7 ml/min, we get good vibrational cooling that is similar to what we obtain with 60 ml/min helium, and above this value, the cooing efficiency saturates as the residual intra‐ion vibrational energy becomes very small. These results of greater nitrogen cooling efficiency per nozzle flow rate are expected based on measured cooling efficiency in laser spectroscopy and supersonic molecular beams experiments.^28^, ^29^ The greater the mass of the SMB cooling gas, the easier it is to accelerate and cool the seeded organic compounds.^28^, ^29^ Briefly, due to lower mass difference between nitrogen and the sample compound, lower number of collisions is needed to accelerate the sample compound to the slower SMB carrier gas velocity and the vibrational cooling per collision is more effective. In addition, nitrogen is a diatomic compound with rotational and vibrational degrees of freedom that further help in its vibrational cooling of sample compounds compared with monoatomic helium. Thus, our initial expectation was that nitrogen as N_2_ will be over seven times more effective than helium in sample compound cooling and this expectation was what we experimentally observed as shown in Figure 1.

**FIGURE 2:**
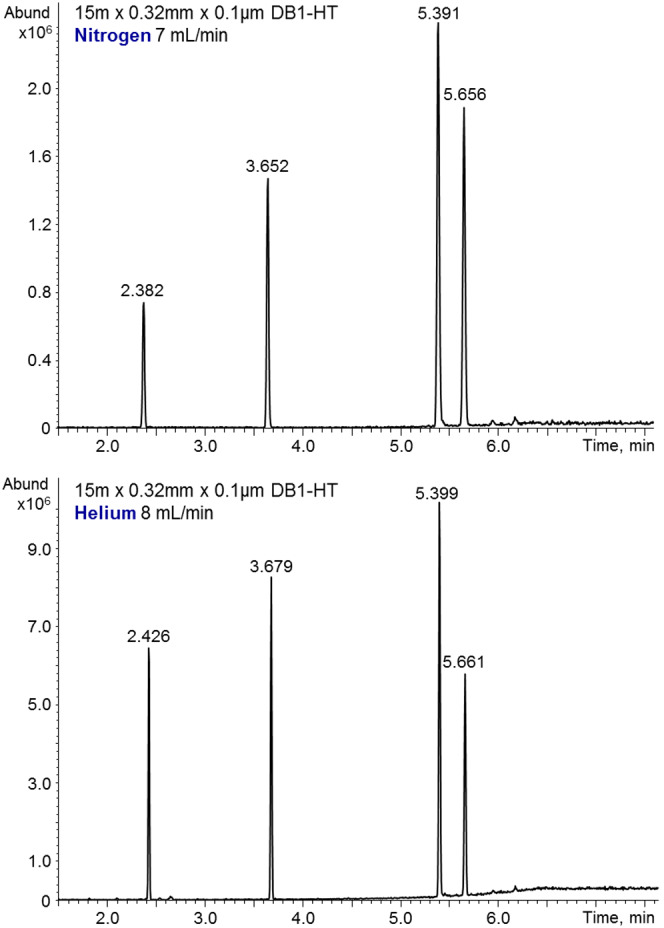
A comparison of test mixture analysis with 7 ml/min nitrogen as carrier gas without any additional make‐up gas (upper mass chromatogram) and 8.0 ml/min helium carrier gas plus 50 ml/min helium as make‐up gas. Note that the nitrogen flow rate was tuned to obtain similar elution times as with helium. The various elution times are indicated.

One of the limitations of nitrogen as GC and GC‐MS carrier gas is that for optimum separation, it slows the chromatographic separation due to slower optimal Van Deemter carrier gas velocity. Contrary to expectations, one can obtain with nitrogen the same elution times as with helium if the chromatography separation requirements are reduced. In GC‐MS with standard EI, there is very little freedom in changing the nitrogen carrier gas flow rate as it adversely affects the sensitivity. Cold EI allows wide change in the nitrogen carrier gas flow rate up to about 8 ml/min with no effect of the nitrogen carrier gas flow rate on the sensitivity. The varied column flow rate is fully compensated by the make‐up gas flow rate and so the supersonic nozzle flow rate is unchanged and consequently the sensitivity is unchanged. Furthermore, the initial few milliliter per minute nozzle flow rate even increases the signal due to improved jet separation. Figure 2 shows the relatively “fast analysis” of our test mixture of 2 ng each n‐C_16_H_32_, methyl stearate, cholesterol, and n‐C_32_H_66_. The same elution times are obtained for the four sample compounds with 7 ml/min nitrogen as with 8 ml/min helium carrier gas flow rate. However, the GC peak widths are broader with nitrogen reflecting the reduced chromatographic separating power.

Table 1 reports the effect of replacing helium with nitrogen (or hydrogen) with respect to GC‐MS full scan peak heights, peak widths, and peak areas for the for test mixture components as shown in Figure 2 (and Figure 5). As shown, the Cold EI peak heights declined with the use of nitrogen for Cold EI in the range of factors of 3 up to 8.7 and larger molecular weight compounds exhibited less signal decline. Furthermore, under column carrier gas flow rate conditions producing the same compound elution times, such as the 7‐ml/min nitrogen and the 8 ml/min helium flow rates, the peak widths increased on the average by a factor of 1.55 in nitrogen. Therefore, the signal peak areas are decreased on the average by a factor of 3.34, which is much smaller than the signal reduction in GC‐MS with standard EI, yet it is with a high (7 ml/min) nitrogen carrier gas flow rate.

**TABLE 1:** A comparison of nitrogen hydrogen and helium data of GC peak heights, peak widths, and peak areas for the test mixture compounds as shown in Figures 2 and 5

Nitrogen and hydrogen vs. helium as the carrier gas										
		*t*_R_[min]			Peak height reduction		Peak width increase		Area reduction	
	Elemental formula, name, and Mol. weight	He	N_2_	H_2_	N_2_	H_2_	N_2_	H_2_	N_2_	H_2_
1	Hexadecane C_16_H_34_ MW = 226.265	2.42	2.38	2.46	8.7	1.1	1.6	0.6	5.3	1.4
2	Methyl stearate C_19_H_38_O_2_ MW = 298.286	3.67	3.65	3.71	5.4	1.4	1.5	0.7	3.4	1.8
3	Cholesterol C_27_H_46_O Mw = 386.354	5.39	5.39	5.43	4.2	1.3	1.6	0.8	2.6	1.7
4	Dotriacontane C_32_H_66_ MW = 450.516	5.66	5.66	5.69	3.0	1.3	1.4	0.8	2.0	1.6
Average					5.3	1.3	1.5	0.7	3.3	1.6

**FIGURE 3:**
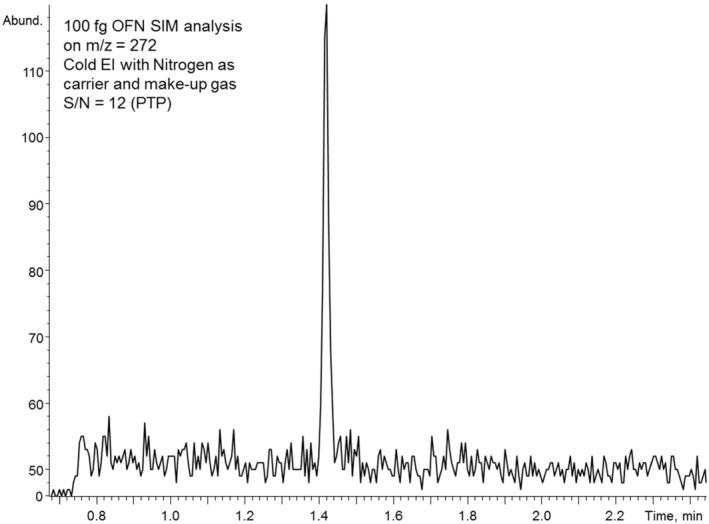
Single ion monitoring mass chromatogram of octafluoronaphthalene (OFN) at 100 fg on‐column amount obtained by GC‐MS with Cold EI using nitrogen as the carrier (3 ml/min) and make‐up gas (5 ml/min)

Because the signal decline in Cold EI with nitrogen compared with the use of helium is moderate, it is interesting to explore the obtained signal‐to‐noise ratio (S/N) in a compound such as octafluoronaphthalene (OFN) that is widely used to demonstrate GC‐MS instrument sensitivity. Figure 3 presents the single ion monitoring (SIM) mass chromatogram obtained with 100‐fg OFN on‐column using nitrogen as both column carrier gas (3 ml/min) and make‐up gas (5 ml/min). The measured OFN signal to noise ratio was 12:1 (peak‐to‐peak), and it was affected by both reduced signal as shown for other compounds in Figure 2 and Table 1 above as well as due to increased noise. The OFN baseline noise increased compared with that with the use of helium carrier gas due to two main reasons: (a) We did not have any carbon black impurities trap in the nitrogen gas line that was connected directly from its cylinder via a 10 m copper gas tubing to the injector and column, and (b) the process of background ion filtration by the zero electric field dual cage fly‐through ion source^27^ is not as effective with nitrogen as with helium because its sample compound acceleration is much lower than with helium.

While the obtained S/N for OFN is significantly lower than with helium,^25^ it is close to what we typically obtain with Agilent 5977 with its Inert standard EI ion source with helium (S/N = 15), and thus, the Cold EI sensitivity with nitrogen is sufficient for standard routine analyses. Furthermore, the Cold EI sensitivity gain versus standard EI is increased, and it is much higher for difficult to analyze compounds,^23^, ^24^, ^25^, ^30^ and this feature is retained with the use of nitrogen in Cold EI.

**FIGURE 4:**
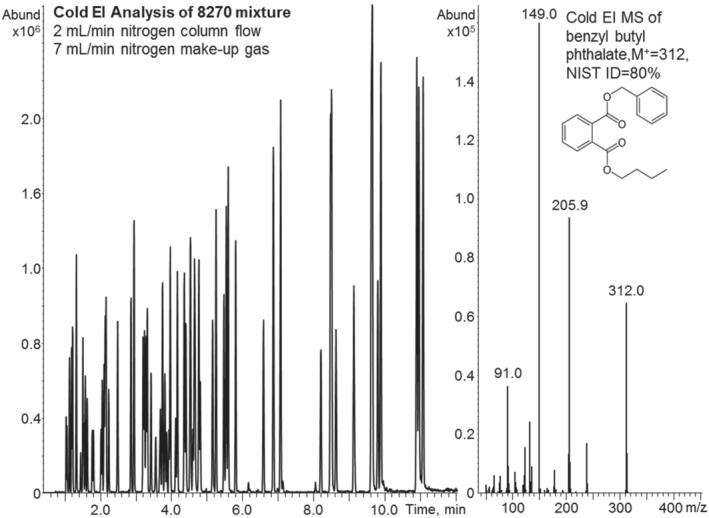
An 8270 semivolatile mixture analysis (Restek 8270 MegaMix part number 31687 with 76 compounds) by GC‐MS with Cold EI using nitrogen as the carrier and make‐up gases at combined flow rate of 9 ml/min (left mass chromatogram). A representative Cold EI mass spectrum of benzyl butyl phthalate (*t*_R_ = 8.21 min) is shown at the right side to demonstrate the effective Cold EI enhancement of the molecular ion that is weak (near absent) in standard EI

In order to demonstrate the applicability of using nitrogen with Cold EI in Figure 4, we demonstrate the USEPA 8270 semivolatile mixture analysis with 76 compounds using nitrogen as the carrier (2 ml/min) and make‐up gas (7 ml/min) and combined nozzle flow rate of 9 ml/min (left mass chromatogram). A representative Cold EI mass spectrum of benzyl butyl phthalate is shown at the right to demonstrate the effective Cold EI enhancement of the molecular ion for this compound that is weak (almost absent) in standard EI. As shown, Cold EI can serve for such general analysis and even provide fast analysis (under 12 min) with 15 m, 0.32 mm I. D column, while using nitrogen as the carrier gas. As shown in Figure 4, there is no peak tailing including for the less volatile late eluters 6 rings PAHs because in Cold EI there is no ion source induced peak tailing in view of its use of contact‐free fly‐through ion source including for the notoriously difficult polar compounds such as pentachlorophenol and benzidine. The performance of GC‐MS with Cold EI in the analysis of USEPA 8270 mixture with helium carrier gas is described in details in Amirav.^31^

### 3.2 GC‐MS with Cold EI operation with hydrogen

**FIGURE 5:**
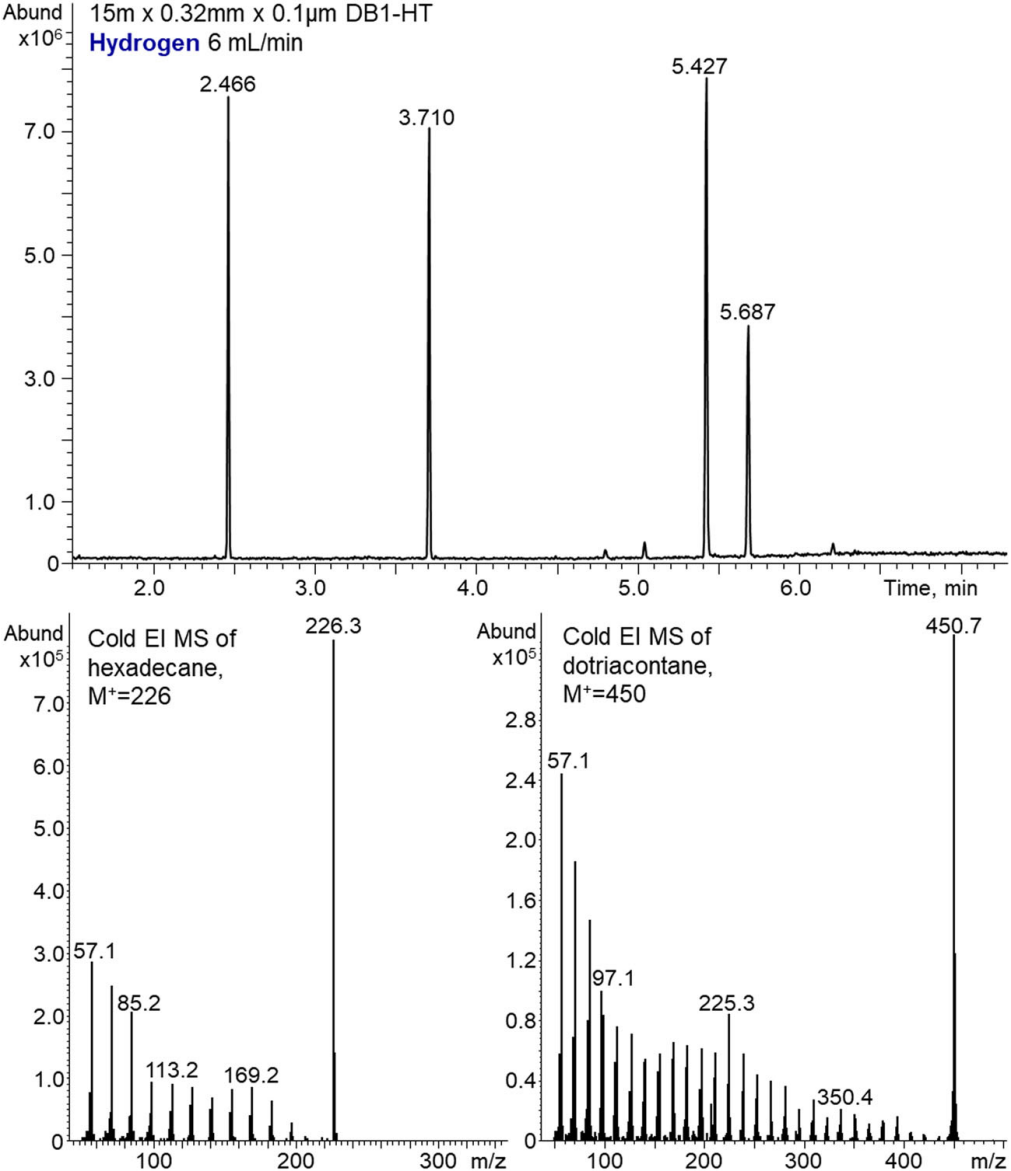
The effect of hydrogen as carrier and make‐up gas on the analysis of a test mixture of 2 ng each hexadecane (n‐C_16_H_32_), methyl stearate, cholesterol, and dotriacontane (n‐C_32_H_66_). The hydrogen carrier gas flow rate was 6 ml/min, whereas the hydrogen make‐up flow rate was 54 ml/min for total hydrogen cooling flow rates from the supersonic nozzle of 60 ml/min. The resulting Cold EI mass spectra of n‐C_16_H_34_ (bottom left) and n‐C_32_H_66_ (bottom right) are shown at the bottom. Note the change in ratio of molecular ion abundance to that of the m/z = 57 fragment ion between the two compounds

The data presented above indicate that nitrogen can serve as a reasonable alternative carrier and make up gas for GC‐MS with Cold EI. However, it is also worthwhile to explore the use of hydrogen as an alternative gas because it is widely available and stable in supply. As with nitrogen, the initial GC‐MS Cold EI experiments with hydrogen were to characterize the vibrational cooling efficiency per given total column and make‐up flow rate. Figure 5 shows the effect of hydrogen as carrier and make‐up gas on the analysis of our standard test mixture of 2 ng each n‐C_16_H_32_, methyl stearate, cholesterol, and n‐C_32_H_66_. The hydrogen carrier gas flow rate was 6 ml/min to produce the elution times similar to those obtained with 8 ml/min helium carrier gas. The hydrogen make‐up flow rate was set as 54 ml/min for total hydrogen cooling flow rates from the supersonic nozzle of 60 ml/min as was used with helium.

The resulting Cold EI mass spectra of n‐C_16_H_34_ (bottom left) and n‐C_32_H_66_ (bottom right) are shown at the bottom. The data indicate that hydrogen can serve as a cooling gas for Cold EI, but its cooling efficiency is lower than of helium, and the bigger the compound the lower the cooling efficiency. While hydrogen provides good cooling efficiency at 60 ml/min nozzle flow rate for hexadecane, its cooling efficiency is noticeably worse for dotiacontane (n‐C_32_H_66_). This is the outcome of greater mass difference between hydrogen and the sample compound that as a result requires more collisions to accelerate the sample compound to the higher hydrogen velocity plus the energy transfer per collision is smaller than with helium. Although increasing the make‐up flow rate to 120 ml/min was possible, safety and turbo pump reliability at such hydrogen flow rate were concerns. The chromatography of the test mixture was improved with narrower peak widths thus better separation with hydrogen as the carrier gas than with helium for similar elution times.

**FIGURE 6:**
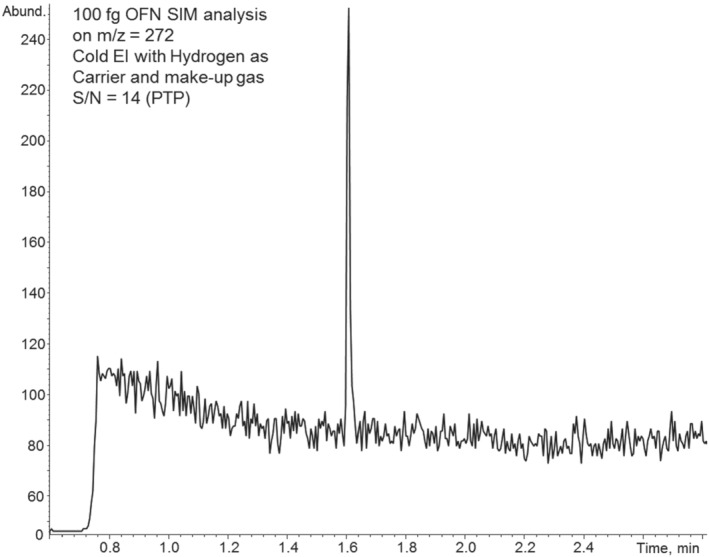
Single ion monitoring mass chromatogram of octafluoronaphthalene (OFN) at 100 fg on‐column amount obtained by GC‐MS with Cold EI using hydrogen as the carrier (2 ml/min) and make‐up gas (58 ml/min)

Figure 6 presents the GC‐MS with Cold EI single ion monitoring mass chromatogram of OFN at 100 fg on‐column using hydrogen as the carrier (2 ml/min) and make‐up gas (58 ml/min). The OFN signal to noise ratio was 14 (peak to peak), which is very comparable S/N (sensitivity) to that obtained with our school of chemistry Agilent 5977 MSD which produced S/N of 15:1 for 100 fg OFN.

We note that while the signal with hydrogen is close to that obtained with helium, we are unable to fully compare it with helium because upon changing helium to hydrogen, the gain of the ion detector was unstable. Initially, the ion detector electron amplification gain increased but subsequently later it reduced, likely due to hydrogen affecting the ion detector surface work function consequently detector electron amplification. We assume that the signal with hydrogen is about 1.6 times lower than with helium (based on experiments with helium after with hydrogen). Additionally, producing the same emission current required a small increase in the filament heating power. However, a significant problem with hydrogen was the resulting high background noise that did not subside in Cold EI even after a few weeks which was inconsistent with the vendor's claims. As shown in Figure 6, the baseline with hydrogen is much noisier (in SIM at m/z = 272 for OFN) than with nitrogen and far noisier than with helium. Despite having higher signal with hydrogen, this high noise is the reason why the OFN S/N in hydrogen is similar to that obtained with nitrogen.

**FIGURE 7:**
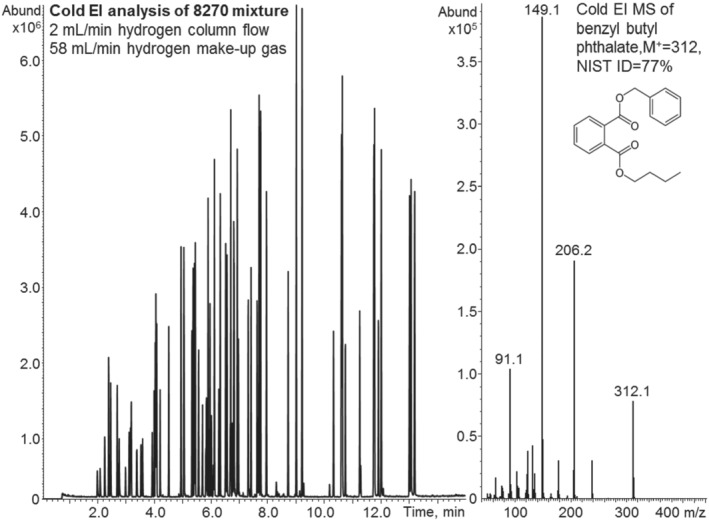
An 8270 semivolatile mixture analysis (Restek 8270 MegaMix part number 31687 with 76 compounds) by GC‐MS with Cold EI using hydrogen as the carrier with 2 ml/min carrier gas flow rate and combined carrier and make‐up gases flow rate of 60 ml/min (left mass chromatogram). A representative Cold EI mass spectrum of benzyl butyl phthalate (*t*_R_ = 10.35 min) is shown at the right side to demonstrate the Cold EI enhancement of the molecular ion that is weak (near absent) in standard EI

Possibly the biggest downside of using hydrogen as the carrier gas is that it is a potentially reactive gas. Figure 7 shows the analysis of the 76 compounds USEPA 8270 semivolatile mixture by GC‐MS with Cold EI using hydrogen as the carrier and make‐up gases at combined flow rate of 60 ml/min (left mass chromatogram). The Cold EI mass spectrum of benzyl butyl phthalate indicates that the Cold EI enhancement of the molecular ion with hydrogen is noticeably lower than with nitrogen (Figure 4) by greater than twofold. This is still far superior to that of standard EI where it is nearly nonexistent.

Reasonable separation of the USEPA 8270 mixture was achieved with hydrogen (Figure 7) and compounds did not display the usual ion source‐related peak tailing due to the contact‐free “fly‐through” ion source. We analyzed the data peak by peak and did not find any degradation or reaction of hydrogen with any of the 76 compounds of the USEPA 8270 mixture and the Cold EI mass spectra have the same fragment ions with hydrogen as with helium or nitrogen.

**FIGURE 8:**
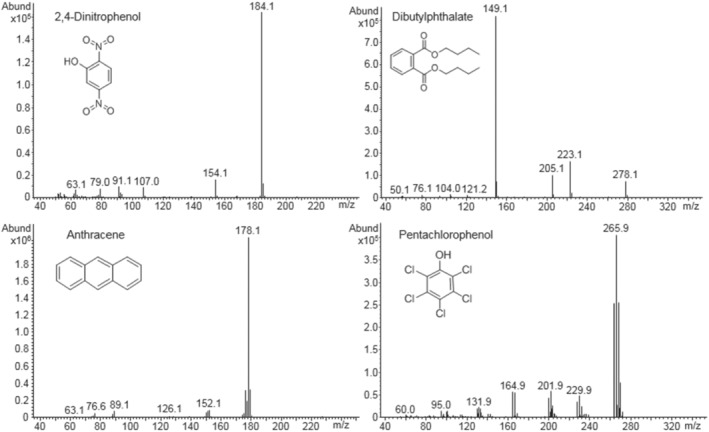
Cold EI mass spectra with hydrogen carrier and make‐up for the selected compounds with names and structures as labeled. No hydrogen induced “breakdown” or conversion is present and all the mass spectral peaks are found in the NIST library

Figure 8 presents four Cold EI mass spectra that were obtained with hydrogen carrier and make‐up gas for the selected USEPA 8270 mixture compounds 2,4‐dinitrophenol, dibutylphthalate, anthracene, and pentachlorophenol. Cold EI is operated with a contact‐free fly‐through dual cage ion source.^27^ Therefore, its generated Cold EI mass spectra are free from any ion source‐related compounds reactions with hydrogen or with the “bare” or “reactive” ion source metal surface that was exposed by hydrogen. The four Cold EI mass spectra of Figure 8 were identified by the NIST library as #1 in its list, and all the generated mass spectral peaks are found in the NIST library with some enhancement of the molecular ions. In contrast, standard EI was found to “poorly” perform with pentachlorophenol and “very poorly” perform (to the extent of not being recommended for hydrogen analysis) with 2,4‐dinitrophenol, dibutylphthalate, and benzyl butyl phthalate^12^ (shown in Figure 7). While we did not expect any ion source reactivity, we were favorably surprised not to find any injector reactivity as reported in the literature.^19^, ^20^ Perhaps the use of a Jennings cup liner, which lacks glass wool in the liner, and high split flows produced little opportunity for reduction. This is an encouraging observation, but it cannot serve to ensure stability for other compounds.

Table 1 compares the chromatographic performance using nitrogen, hydrogen, and helium in terms of several metrics. GC‐MS with Cold EI can produce very similar elution times via tuning the carrier gases flow rates, which were 8 ml/min with helium, 6 ml/min with hydrogen, and 7 ml/min with nitrogen. The average peak height reduction was 5.3 with nitrogen, whereas with hydrogen, the average peak height reduction factor was smaller at 1.3. The peak width was increased by a factor of 1.5 with nitrogen and reduced by a factor of 0.7 with hydrogen thus the average signal (peak area) reduction factors were 3.3 with nitrogen and 1.6 with hydrogen. However, the noise was greater with hydrogen and thus the LOD losses were similar with hydrogen and nitrogen as observed with OFN in Figures 3 and 6.

## 4 CONCLUSIONS AND DISCUSSION

Helium remains the carrier gas of choice even if purchasing and holding additional spare helium gas cylinder(s) is required if one expects short‐time interruption/shortage periods. However, if an unavoidable helium shortage occurs, these results illustrate a viable solution for continued GC‐MS with Cold EI operation via the use of nitrogen instead of helium. Unlike with GC‐MS with standard EI, nitrogen can be used with Cold EI using the same capillary column as in helium, thereby making system reconfiguration avoidable. Due to Cold EI's flexibility in column flow rates, the compound retention times can be preserved with nitrogen, simplifying analytical concerns (no changes in databases, quantitation tables, etc.) with a small sacrifice due to slightly increased peak widths. Since switching from helium to nitrogen and back is as simple as closing or opening the helium and nitrogen input valves (illustrated in the graphical abstract) and because the equilibration time is very rapid (only a few minutes), downtime is negligible. This gas input change can be automated by software control and becomes as simple as loading an acquisition method unlike the manual values used here for proof of concept.

Nitrogen is very effective in providing a high degree of vibrational cooling, and at 7–8 ml/min combined column carrier and make‐up flow, nitrogen is as effective for vibrational cooling as 60 ml/min helium. The column flow rate can be changed or programmed with nitrogen up to 8 ml/min without affecting the signal (unlike in standard EI), and exceeding this value is possible but with some further loss of signal. On the whole, GC‐MS with Cold EI can be effectively operated with nitrogen as the column carrier and make‐up gas with minimal changes in the analysis method. Still, the use of helium is preferred over nitrogen as it provides higher sensitivity, lower LOD, better separation (or faster analysis), and possibly greater range of compounds amenable for analysis via the use of high (up to 100 ml/min) column flow rate.^23^, ^24^, ^25^, ^30^

Cold EI operation with hydrogen provides the benefit of somewhat improved chromatography. GC‐MS with Cold EI does not produce as many issues with chemical reactivity for the USEPA 8270 mixture compounds studied here in part due to the Cold EI fly‐through ion source design, which lacks surface collisions and long compound‐ion volume residence times of typical GC‐MS with standard EI ion sources as so produces superior compound spectral and response stability. In terms of safety, there is clearly a preference for the use of nitrogen over hydrogen. Cold EI requires over 60 ml/min hydrogen flow rate plus whatever the injector requires with its split flow rate and thus can require over 100 ml/min hydrogen, which is a substantial and perhaps unsafe amount. In addition, hydrogen suffers from high baseline noise, which appears a permanent feature. Unlike hydrogen, which requires high upfront costs to acquire a generator and continued service costs or special regulators (SS diaphragm) and complex safety compliance requirements, nitrogen requires only having a one cylinder, regulator, and filter that can serve well for years as a backup to helium for the hopefully very rare cases of helium supply interruption.
